# Proposal of a New Method for Measuring Förster Resonance Energy Transfer (FRET) Rapidly, Quantitatively and Non-Destructively

**DOI:** 10.3390/ijms131012367

**Published:** 2012-09-26

**Authors:** Paul Johannes Helm

**Affiliations:** Center of Molecular Biology and Neuroscience and Institute of Basic Medical Sciences, Department of Anatomy, University of Oslo, P.O. Box 1105-Blindern, NO-0317 Oslo, Norway; E-Mail: p.j.helm@medisin.uio.no; Tel.: +47-228-51159; Fax: +47-228-51499

**Keywords:** FRET, LSM, dynamic, non-destructive, electro-optic modulator, beat, saturation, modulation

## Abstract

The process of radiationless energy transfer from a chromophore in an excited electronic state (the “donor”) to another chromophore (an “acceptor”), in which the energy released by the donor effects an electronic transition, is known as “Förster Resonance Energy Transfer” (FRET). The rate of energy transfer is dependent on the sixth power of the distance between donor and acceptor. Determining FRET efficiencies is tantamount to measuring distances between molecules. A new method is proposed for determining FRET efficiencies rapidly, quantitatively, and non-destructively on ensembles containing donor acceptor pairs: at wavelengths suitable for mutually exclusive excitations of donors and acceptors, two laser beams are intensity-modulated in rectangular patterns at duty cycle *½* and frequencies *f*_1_ and *f*_2_ by electro-optic modulators. In an ensemble exposed to these laser beams, the donor excitation is modulated at *f*_1_, and the acceptor excitation, and therefore the degree of saturation of the excited electronic state of the acceptors, is modulated at *f_2_*. Since the ensemble contains donor acceptor pairs engaged in FRET, the released donor fluorescence is modulated not only at *f*_1_ but also at the beat frequency Δ*f*: = |*f*_1_ − *f*_2_|. The depth of the latter modulation, detectable via a lock-in amplifier, quantitatively indicates the FRET efficiency.

## 1. Introduction

In 1923, based on theoretical considerations by Klein and Rosseland [1], Franck and Cario described their observation that energy stored in the electronic configuration of excited gas atoms of one type can be transferred to gas atoms of another type, given that the amount of energy stored in the excited gas atoms of the first type is suitable for the excitation of the gas atoms of the second type [2]. They concluded from their experiments that the transfer of kinetic energy during the atomic collisions of the second kind, which at the same time resulted in the transfer of quantum energy, can—if required—cover missing quantum energy for the previously-described energy transfer from gas atoms of one type to the gas atoms of a second type.

Jean Perrin suggested that the transfer of the aforementioned quantum energy could be explained as a process he described as molecular induction, *i.e*., an energy transfer not mediated by fluorescent light, or more simply, a radiationless energy transfer [3].

The phenomenon has been studied in detail during the decades since and termed “Electronic Energy Transfer” [4], “Electronic Excitation Energy Transfer” [5], or—incorrectly—“Fluorescence Resonance Energy Transfer.” Since the late 1950s, the terms “donor” and “acceptor” have been used to describe the atoms or molecules participating in the process [4].

The process occurring, the luminescence of the acceptor instead of that of the donor is observed even if it is the donor—and not the acceptor—which is, or has been, stimulated by means of an external energy source.

Th. Förster interpreted the phenomenon quantitatively and in quantum mechanical terms [6]. It is today commonly referred to as the “Förster Resonance Energy Transfer”, abbreviated “FRET”. He concluded in mathematical terms that, other than during the energy transfer via a mediating luminescent photon, or radiative energy transfer, FRET only can occur if the two chromophores are quasi-co-localized and he derived that the rate of the energy transfer by means of FRET is dependent on the sixth power of the distance between donor and acceptor [6,7]. Arnold and Oppenheimer confirmed the result [8].

Förster’s theory has been verified experimentally by Lubert Stryer and Richard P. Haugland, who realized that the phenomenon could be used to reliably measure distances on the molecular scale and pertinently characterized the method as a “spectroscopic ruler” [9].

The distance between a pair of chromophores suitable for FRET that yields an efficiency of 0.5 has been named the “Förster radius”. It is a characteristic value for any FRET pair of chromophores.

FRET has become a standard method in a number of fields, such as fluorescence microscopy. Bioluminescent dye substances suitable as tracers in biological cells feature Förster radii between approximately 10 Å and 100 Å. Quantitative interpretation of FRET efficiencies in suitable regions of interest (ROI) in digitized microscopic images is equivalent to measuring relative or, given a sufficient calibration, even absolute distances in domains surpassing the resolution limit of conventional or even confocal light microscopes by up to two orders of magnitude. Thus, FRET indirectly extends the resolution of the light microscope into domains that would otherwise be accessible only by electron microscopy, and establishes a powerful tool for co-localization studies on live specimens. By way of example, it is possible with FRET to observe a variety of cellular processes involving protein-protein interactions, both *in vitro* and *in vivo* [10]. Since the late 1990s, FRET has developed into a popular method for recordings of ion concentration, such as [Ca^2+^] (***r***, *t*) measurements [11–13]. Dynamic FRET measurements on living specimens with the goal of changing donor-acceptor configurations, have been done both semi-quantitatively [14] and fully quantitatively by means of “Fluorescence Lifetime Imaging Measurements” (FLIM) [15–18].

However, the quantitative interpretation of microscopic FRET images is still cumbersome. A number of methods have been developed for the purpose. Pietraszewska-Bogiel and Gadella [19] provide a plain and concise review; Ishikawa-Ankerhold *et al*. [20] give a more comprehensive review. The most relevant techniques in use include intensity-based FRET methods [21,22], the spectral FRET method [23,24], acceptor depletion FRET methods [25–28], the donor photo bleaching FRET method [29,30], the FRET FLIM methods [15–18], and the anisotropy FRET method based on the—long-known [31,32]—polarization dependence of the FRET process [33]. Recently, Xie *et al*. reported the development of two new approaches improving FRET determinations exclusively based on intensity measurements [34].

The aim of this proposal is to present a new approach for fully quantitative, rapid, and non-destructive FRET measurements.

## 2. Description of the System

### 2.1. Principle of Operation

**Figure 1:**
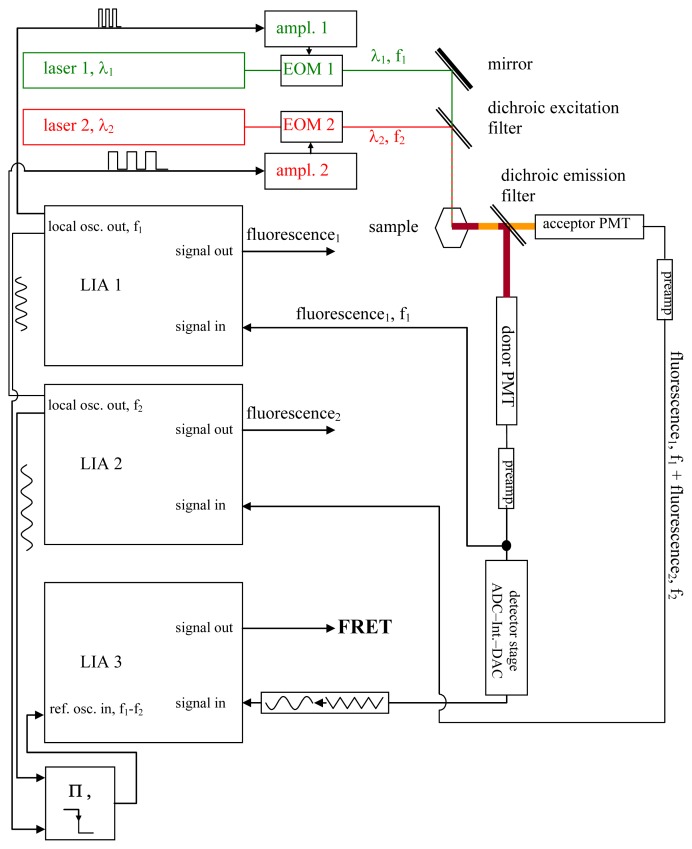
The setup. In this figure, the setup required for the Förster Resonance Energy Transfer (FRET) measurements is shown; it is described and explained in detail in the text (see section 2.1.). While imaging systems such as laser scanning microscopes will require modulation frequencies in the megahertz range and consequently demand the use of EOMs—which, for their part, more or less enforce the use of lasers as light sources—classical light sources combined with band-pass filters and suitable chopper wheels as modulators may instead be used in applications not needing rapid data acquisitions.

**Figure 2:**
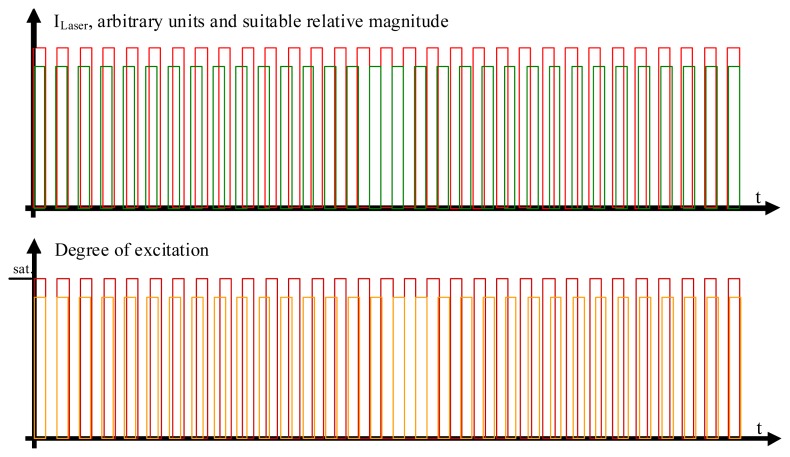
The laser beam excitations and degrees of modulation. In the upper part of the figure, the rectangle-shaped modulation pattern of the two laser beams is shown. The modulation frequency of the laser light exciting the acceptor chromophores, colored in red, is slightly different from the modulation frequency of the laser beam exciting the donor chromophores, colored in green. The respective degrees of excitation of the acceptor molecules (deep red color) and donor molecules (colored in orange) are shown in the lower part of the figure. While it helps to understand the molecular processes in qualitative terms if one first assumes that the acceptor beam is so strong as to completely saturate the excited state in all acceptor molecules in the measurement volume, it is not necessary that the laser beam exciting the acceptor molecules in fact be so strong that it effects a degree of excitation of the ensemble of the involved acceptor molecules close to saturation, as will be shown in the quantitative analysis.

Using a somewhat modified and supplemented hardware arrangement of an “IMS-technique setup” [35–42], it may be possible to perform rapid, quantitative, and non-destructive FRET-measurements (see Figure 1). A laser beam at a wavelength λ_1_, which should be suitable for donor excitation while simultaneously exciting the acceptor as little as possible, is used to excite the donor, while the acceptor is excited by another laser beam at λ_2_, whose wavelength should likewise excite the donor as little as possible. Each of the two laser beams is modulated in a rectangular pattern at duty cycle *½* (see Figure 2) at the respective frequencies *f*_1_ and *f*_2_ by means of Electro-Optic Modulators (EOM). Neither *f*_1_ nor *f*_2_ have to attain values that would be considered large, compared to what can be achieved by means of decent commercial EOM amplifiers. Thus, the rectangular shape of the intensity modulations of the laser beams will not be noticeably distorted at the edges. The requirements are that the modulation frequencies, (A), are large compared to Δ*f*: = |*f*_1_ − *f*_2_|; (B) are large compared to the speed at which the processes to be studied by the FRET phenomenon are occurring; and (C) are large compared to the sampling frequency of the data acquisition, which in case of imaging systems are the inverted pixel integration times. Δ*f* is the frequency of the beat generated by the oscillations at *f*_1_ and *f*_2_. Additionally, the respective oscillation periods T_1_ and T_2_ must be long compared to the lifetimes of the excited states of the chromophores. 15 MHz and 12 MHz as *f*_1_ and *f*_2_, and thus 3 MHz as Δ*f* (given Δ*f* = |*f*_1_ − *f*_2_|), are suitable even for pixel sampling times in the microsecond region. Modulation of the laser beam effects the modulation of chromophores’ electronic states (see Figure 2).

The signals needed to modulate the laser beams are generated by the local oscillators of two lock-in amplifiers (LIA) LIA1 and LIA2, and are fed as modulation signals at *f*_1_ or *f*_2_ into the EOM amplifiers. They are also fed into the reference local oscillator input of a third LIA, LIA3, via a mixer stage. LIA3, is therefore frequency- and phase-locked to the beat at Δ*f*.

A dichroic emission filter spectrally separates donor fluorescence and acceptor fluorescence, which are detected by means of photomultiplier tubes (PMTs). If required, additional band pass filters (not shown in Figure 1) can be inserted between the dichroic emission filter and the PMTs in order to minimize fluorescence cross talk between the two channels.

The fluorescence signal from the acceptor is detected by a photomultiplier tube (the acceptor PMT) converted to voltage by its pre-amplifier, and fed into LIA2.

The donor fluorescence is also recorded by means of the donor PMT and converted to a voltage signal by means of a pre-amplifier. This voltage signal is registered by means of LIA1.

**Figure 3:**
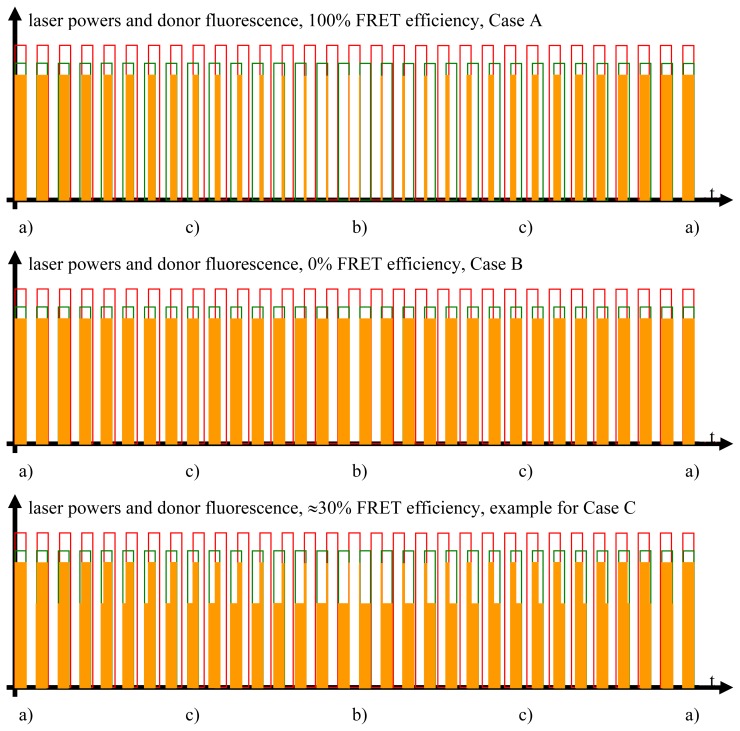
Donor fluorescence as a function of the FRET efficiency. The FRET efficiency is detected via the donor fluorescence. In this figure, the donor fluorescence, symbolized by the orange rectangles, is shown as a function of the time and the FRET efficiency. Unaltered donor fluorescence is only generated when there is solely donor excitation with no simultaneous FRET. Depending on the FRET efficiency, a certain fraction of the excitation energy stored in the excited donor molecules is not emitted as fluorescence but radiationlessly transferred to the acceptor. This is the reason for the shapes of the “geometrically distorted” orange rectangles in the case of the example of 30% FRET efficiency (see text for detailed explanation).

**Figure 4:**
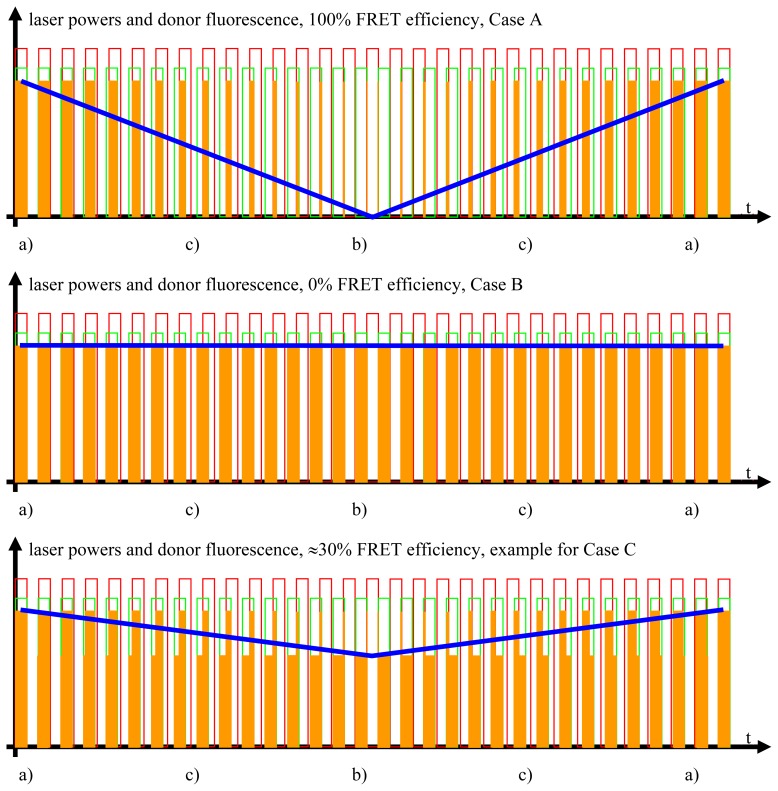
The FRET signal. The blue curves show the donor fluorescence as measured by the donor photomultiplier tubes (PMT) and filtered by the detector stage. The blue curve contains all the information necessary for the quantitative FRET analysis.

As explained in later figures and paragraphs, the donor fluorescence is not modulated solely by *f*_1_. Instead, whenever the donor and acceptor are FRETing, there also exists a triangle-shaped modulation of the donor fluorescence at Δ*f* = |*f*_1_ − *f*_2_| (see sections 2.3., 2.4. and Figures 2–4). The depth of this modulation is directly dependent on the actual FRET efficiency (as will be explained in detail later in the text). In order to register this modulation depth, a detector stage consisting of an analog digital converter (ADC), an integrator, and a digital analog converter (DAC) is used to pre-process the total output from the donor PMT. The triangle-shaped, slow modulation pattern, after having been transformed into a sinusoidal pattern, is subsequently registered by LIA3. LIA3 is frequency- and phase-locked to the beat signal at Δ*f* by means of the “local oscillator out” signals of LIA1 and LIA2, as well as by a multiplier and a low-pass filter. Thus, signals at Δ*f* are detected. As explained in the text and the other figures, the output signal provided by LIA3 allows for quantitative determination of the FRET efficiency.

While it is not necessary for the determination of the FRET efficiencies to actually measure the outputs from LIA1 and LIA2, these outputs are nevertheless essential for the practical purpose of adjusting the optimal phases in LIA1 and LIA, which are required as input to lock LIA3 to the beat.

### 2.2. A Suitable Test Preparation

A suitable test preparation is, in the simplest case, a solution of a substance that consists of a FRET donor chemically bound to a FRET acceptor. Depending on which FRET conditions are to be simulated, a certain number of “spacers” may be “inserted” to separate donor and acceptor and keep them at a constant mean distance. It is technically possible to synthesize both “donor-acceptor” molecules with ideal FRET-conditions as well as “donor-spacer-acceptor” groups with spacers of well-defined lengths to simulate different degrees of the FRET efficiency. The synthesis and successful use of a suitable substance has been described by Stryer and Haugland [9].

### 2.3. Processes on the Molecular Level

#### 2.3.1. Case A, Ideal FRET Conditions, Donor and Acceptor Close to Each Other, Optimal FRET

Donor and acceptor are FRETing whenever possible, *i.e.*, when the acceptor has not been excited by the laser beam at λ_2_ right ahead of the moment, when the donor can transfer the energy to the acceptor. Since both modulation frequencies are nearly identical, the phase difference between both rectangular modulations will oscillate at the beat frequency. Thus, the FRETing also oscillates at the beat frequency. A stronger laser beam power at λ_2_ will effect a larger amplitude of this oscillation and therefore a larger degree of excitation of the involved acceptor molecules. In the limiting case, all the acceptor molecules will be in the excited state.

Studying the situation in detail, one has to consider three cases (compare Figure 2 and sectors (a)–(c) in the graphs of Figures 3 and 4)

- Donor excitation and acceptor excitation are close to in phase.
  In this case, the donor fluorescence as well as the acceptor fluorescence are modulated at either *f*_1_ or *f*_2_, at which the modulation will be quite deep. There are strong signals in LIA1 and in LIA2 before the respective amplifier stage. In this simplified analysis it is assumed that during (a) and those parts of (c) (see below) when the direct excitation of the acceptor by the laser beam at λ_2_ and FRET excitation of the acceptor are competing, laser excitation is dominantly strong. A quantitative analysis is provided later on in this article.
- Donor excitation and acceptor excitation are close to entirely counter phase.
  In this case, the acceptor is fluorescing all the time, since it is excited all the time, either by FRET or by the laser beam at λ_2_. Donor fluorescence is close to zero at all times, since the donor excitation energy is transferred to the acceptor. The modulation depths of both donor and acceptor fluorescence will be small. There will not be any considerable signal in either LIA1 or LIA2 before the respective amplifier stages.
- All intermediate states.
  (a) and (b) will be present, each during its respective part of the oscillation period. The average value of the donor fluorescence—integrated over many modulation periods—will oscillate at the beat frequency. The envelope of this beat oscillation is a triangular signal, which LIA3 registers if the signal by the donor fluorescence PMT has been pre-processed by a detector stage/“ADC-Integrator-DAC” circuit prior to de-modulation.

#### 2.3.2. Case B, Ideal Non-FRET Conditions, Donor and Acceptor Far Enough from Each Other to Render Any FRET Process Impossible

Here, LIA3 will not detect anything; the LIA3 output is zero. Note that the signals of both PMTs are not directly mixed. Even if the total fluorescence still shows a strong beat signal generated exclusively by the two directly excited fluorescence patterns, “dichroic mirror 2” plus eventual band pass filters (see Figure 1) separate the signals at both oscillations, *f*_1_ and *f*_2_.

#### 2.3.3. Case C, Any Intermediate Situation

The output signal of LIA3 will be of intermediate strength.

### 2.4. Qualitative Analysis of FRET Measurements

The following description is based on the reasonable assumption that the periods of the modulation patterns are long compared to the optical relaxation times of the chromophores. Moreover, the difference between the two frequencies is assumed to be small compared to the frequencies themselves. At the same time, both, the periods of the individual modulation frequencies and the period of the frequency difference should be sufficiently short compared to the pixel sampling times. 15 MHz and 12 MHz as *f*_1_ and *f*_2_, and thus 3 MHz as Δ*f* (given Δ*f* = |*f*_1_ − *f*_2_|) are suitable even for pixel sampling times in the microsecond region.

**Figure 5:**
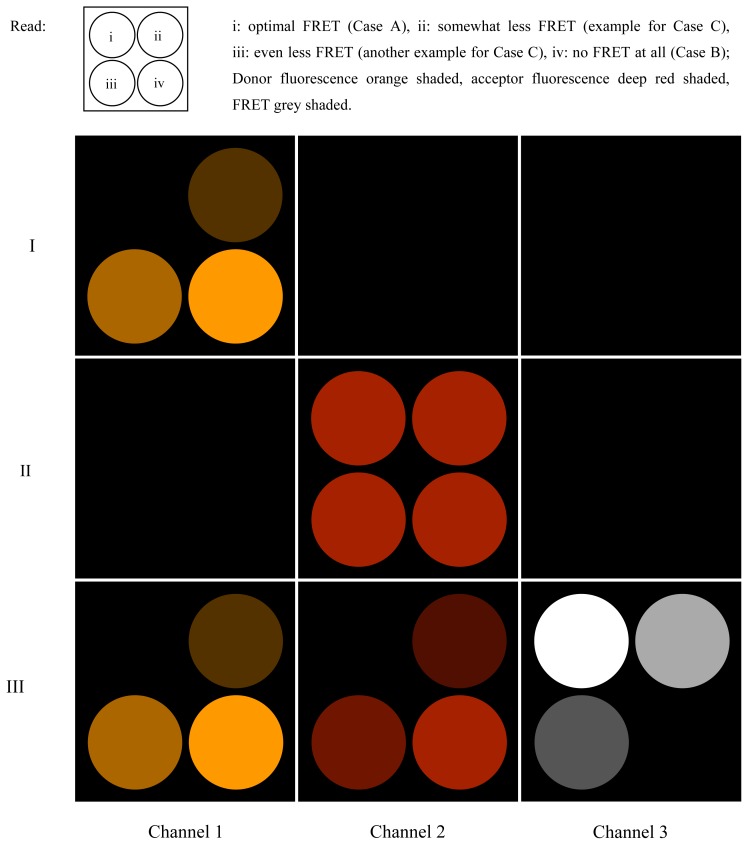
FRET on a microscopic test preparation. The image of a hypothetical test preparation is shown when scanned as a quadratic frame and illuminated with different excitation wavelengths. Four circular regions in the scanned field contain uniform specimens with different FRET conditions. Rows I to III show the result of illuminations with, respectively, λ_1_ only, λ_2_ only, and simultaneously with both λ_1_ and λ_2_, at which the respective laser beams are modulated as outlined in the text. Channel numbers are related to the LIA numbers as defined in Figure 1 and in the text.

In a genuine sample for laser scanning microscopy, the different cases (A–C, see above) will usually be realized locally in the preparation (possibly pixel-wise, for reasons of clarifications as ROIs in Figure 5). Depending on whether there is any FRET or not, and in case of FRET, depending on the FRET efficiency, the pixel values generated by LIA3 detection are smaller or larger. When performing a scan, the signals from the three LIAs will produce three image frames. Channels 1 and 2 are directly acquired. The analog signals from the donor PMT and LIA1 as well as from the acceptor PMT and LIA2—respectively the averages of their beat values—are registered via the ADC of a Laser Scanning Microscope (LSM).

Channel 3, however, exclusively represents the degree of FRET. In other words, the more efficient FRET is in the respective pixel location, the larger the respective pixel value will be.

The entire measurement process can be developed in three stages (described below), of which, strictly speaking, only the third is truly necessary to determine the FRET efficiency (see Figure 5):

- At first, the preparation is illuminated solely by (λ_1_, *f*_1_), and the phase on LIA1 is optimized to attain the largest possible value in Channel 1 (the part of the donor fluorescence that is not “FRETed away”). Dependent on the degree of FRET, the signal will be weaker or stronger in Channel 1 (see i, ii, iii, iv). The FRET fluorescence of the acceptor cannot be seen in Channel 2 since it does not oscillate at *f*_2_ during this step, but at *f*_1_ instead. The signal is zero in Channel 3.
- Now, the preparation is illuminated solely by (λ_2_, *f*_2_). LIA2 is optimized for directly-excited (*i.e.*, non-FRET) acceptor fluorescence. All the regions (i, ii, iii and iv) will shine with equal intensity, since the acceptor is being excited directly and with modulation frequency *f*_2_. Channels 1 and 3 are entirely dark.
- Finally, the preparation is illuminated simultaneously by (λ_1_, *f*_1_) and (λ_2_, *f*_2_). In region i, the donor fluorescence in Channel 1 is zero. The reason for this is that donor and acceptor are FRETing. The value of Channel 2 in i is zero since the modulation depth of the acceptor is zero.

In situation III, wherein both laser beams are simultaneously active, regions i in Channels 1 and 2 will in practice not be perfectly zero. FRET efficiency never completely attains 100%, so a small fraction of rest donor fluorescence will remain at *f*_1_. Additionally, depending on the strength of the laser beam at (λ_2_, *f*_2_), there will unavoidably be a rest modulation depth of acceptor fluorescence at *f*_2_.

In Channel 3 i, the signal is strong since the beat amplitude is large (beat frequency must be so large that its period is short compared to the pixel dwelling time). In ii, iii, and iv, the FRET efficiency is decreasing more and more.

By means of this method, the FRET efficiency can be measured quantitatively and independently of the optical decay times of the chromophores. Of course, the shorter the acceptor decay time is, the larger the power of the laser beam at (λ_2_, *f*_2_) must be in order to attain saturation of the excited state in the acceptor molecules. A pixel value close to 255 in an 8-bit instrument, and a pixel value close to 4095 in a 12-bit instrument indicate 100% FRET efficiency; pixel value zero indicates 0% FRET-efficiency. The *quantitative* approach in terms of formulas is presented in the next paragraph.

### 2.5. The Quantitative Analysis of FRET Measurements

The method so far presented in qualitative terms is entirely based on the beat of the donor fluorescence. This beat is generated by exposing the acceptor molecules to an *f*_2_-modulated laser beam at the proper excitation wavelength λ_2_. By means of this laser beam, the capacity of the acceptor molecules to participate in FRET is modulated.

The minimum value of the beat in the donor fluorescence is generated when there is no direct excitation of the acceptor molecules while the donor excitation is at its maximum—it is, realistically, assumed that the optical relaxation times of the chromophores are short compared to periods of the oscillations at *f*_1_ or *f*_2_. Consequently, the minimum value of the beat in the donor fluorescence is exclusively dependent on the donor excitation and the mutual distance between the donor and acceptor molecules. Thus, this measurement value is trustworthy.

Some initial doubts could possibly be expressed concerning the trustworthiness of the maximum value of the beat in the donor fluorescence. This value, it seems at first, can exclusively be measured while the direct acceptor excitation is so dominant that all the acceptor molecules are in the excited state when the donor molecules are ready to start a FRET process. In other words, the direct excitation has to completely dominate the FRET excitation. For this purpose, Laser 2 has to drive the excitation of the acceptor molecules into saturation. Even if this is technically possible and even if modern dye substances are relatively resistant against bleaching, a biological preparation could possibly suffer from this treatment. Moreover, from a purely esthetical point of view, this appears to be a somewhat brute force approach.

However, there is a solution to this problem:

First, the situation can be optimized by performing two pre-experimental measurements aimed at the proper adjustment of the powers of the two laser beams, which must be set individually so that the excitations of the donor or acceptor molecules are just attaining saturation. This setting of the powers is attained by observing the increase of the individual fluorescence signals. Exceeding the value for excitation saturation results in a reduction of the signal passing the LIAs, since the excitation modulation depth attainable by EOM modulation will decrease. Thus, the amplitude of the beat to be measured later is maximized. In order to obtain this measurement, it is advantageous to prepare some region on the cover slip or in the Petri dish close to the preparation with a small quantity of test substance with known FRET efficiencies NULL and ONE. In most cases, doing so will not present any practical problem.

Second, the idea is that the amplitude of the beat of the donor fluorescence always is a quantitative relative FRET scale. This is true even if the two extremes in the donor fluorescence beat (*i.e*., the amplitude of that oscillation) do not express the difference between “complete FRET” and “zero FRET,” but rather the difference between “practically strongest attainable FRET” and “rest FRET”. This will be proved mathematically:

Let *F* be the FRET efficiency. Then

(1) $$0\leq F=1-\frac{M}{N}\leq 1$$

where *N* is the hypothetical maximum of the donor fluorescence (no acceptor molecule is accepting FRET energy) and *M* is the donor fluorescence when FRET is occurring.

What is happening when there is induced a direct acceptor excitation by means of Laser 2, in mathematical terms, is a reduction of the FRET efficiency. If the power of Laser 2, *J*(λ_2_), is increasing, M is also increasing. In the limiting case of an infinitely large *J*(λ_2_), *M* approaches *N*,

(2) $$\underset{J(\lambda_{2})\to \infty }{\text{lim}}\;M(J(\lambda_{2}))=N$$

The problem now is to establish a functional relationship among *M*, *N*, and *J*(λ_2_). Considering the conditions for *J*(λ_2_) at zero or at maximum, the following formula, fulfilling the aforementioned conditions by means of an exponential function, is suggested:

Define *M*_0_:=*M*(*J*(λ_2_)=0), then

(3) $$M(J(\lambda_{2}))=M_{0}+{\text{e}}^{-\frac{1}{J(\lambda_{2})}}(N-M_{0})$$

Equation 3 into Equation 1 renders for the FRET efficiency as a function of the power of the laser exciting the acceptor molecules

(4) $$F=1-\frac{M_{0}+{\text{e}}^{\frac{1}{J(\lambda_{2})}}(N-M_{0})}{N}$$

In the limiting case of *J*(λ_2_) = 0, this equation is identical to the definition of the FRET-efficiency (see above), while the FRET-efficiency converges to zero, when *J(*λ_2_) increases to large values. Thus, the boundary conditions are fulfilled.

The actual measurement value, as delivered by LIA3, is the modulation depth for the FRET signal oscillation, *i.e.*, the amplitude of an envelope of the beat signal

(5) $$A=k\cdot (M(J(\lambda_{2}))-M_{0})$$

where *k* is a proportionality factor.

Assuming two locations in a preparation with different FRET efficiencies *F*_1_ and *F*_2_, the ratio of these efficiencies is

(6) $$\frac{F_{1}}{F_{2}}=\frac{N-M_{0_{1}}}{N-M_{0_{2}}}$$

where (see above) it is assumed that *N*_1_ and *N*_2_ are identical and are named *N*.

What do the ratio of the LIA3 signals at the two places look like? Inserting the respective terms in the definition equations, Equation 3 into Equation 5, yields:

(7) $$\frac{A_{1}}{A_{2}}=\frac{k\cdot \left[{\text{e}}^{-\frac{1}{J(\lambda_{2})}}\cdot (N-M_{0_{1}})+M_{0_{1}}-M_{0_{1}}\right]}{k\cdot \left[{\text{e}}^{-\frac{1}{J(\lambda_{2})}}\cdot (N-M_{0_{2}})+M_{0_{2}}-M_{0_{2}}\right]}=\frac{N-M_{0_{1}}}{N-M_{0_{2}}}$$

which, because of Equation 6, means

(8) $$\frac{F_{1}}{F_{2}}=\frac{A_{1}}{A_{2}}$$

Thus, the relative FRET efficiencies at different locations in the preparation are directly related to the amplitudes (*i.e.*, the pixel values) in the LIA3 image at these places.

If one has a location in the preparation with a microscopic drop of a test substance with a known absolute FRET efficiency, the absolute FRET efficiency at all other locations in the same preparation can be determined by the relative quantification shown above. The same can, of course, be done by means of separate test preparations, for which the FRET efficiency is known. The only demand is that the laser effect be unchanged between the experiment and the control measurements.

The system therefore allows for fully quantitative and dynamic measurements. Given this, it is not necessary to saturate the excited states of all the acceptor molecules in the measurement volume by means of an exceedingly strong laser beam.

It should be mentioned that the quantitative analysis of FRET images opens the dynamics of intracellular [Ca^2+^] at confocal spatial resolution for quantitative measurements, since FRET-based [Ca^2+^] indicators are now available [12,13].

### 2.6. FRET and Non Linear Laser Scanning Microscopy

Common commercially available tunable titanium sapphire lasers have pulse rates of approximately 80 MHz.

Using one or two phase modulating EOMs, a beat situation could be simulated. The phase modulation would accelerate or retard one or both of the laser beams. Thus, a beat can be simulated by electronic means, provided two titanium sapphire lasers are available and the two chromophores in the respective donor acceptor pair can be excited selectively, each chromophore by one of the beams. The method then could for example, be used for semi-quantitative or even quantitative [Ca^2+^] measurements employing the “Cameleon” dye, with which CFP and YFP can be excited selectively by specific Ti-Sap laser wavelengths [12,13].

### 2.7. Multiple FRET Processes

The system can be extended to include appropriate numbers of laser lines, EOMs, PMTs, and LIAs, in order to quantitatively determine competing FRET processes in one sample. In this case, several different donors interact with one acceptor or one donor interacts with several different acceptors. It is essential that there be available illumination and detection wavelength bands for the involved chromophores, which must not cause too much mutual cross-excitation or cross-channel detection. In a situation where for example, there would be one donor interacting with three different acceptors, one laser line and EOM would be needed for the donor and one laser line and EOM would be needed for each acceptor, in addition to one LIA for each acceptor and one LIA for each FRET process, requiring altogether four laser lines, four EOMs, three PMTs, and seven LIAs.

While it is not a problem to understand that the method can be used in situations, where, for example, each of the donor molecules interacts with an acceptor of either the first, second, or third type, the method would even allow determination of how much FRETing would occur between the donor and several acceptors if, in an ensemble, a large fraction or even all of the donor molecules could enter FRET processes with two acceptors, at which the donors would be in FRET range distance to acceptors molecules of both types at the same time, *i.e*., triangular co-localization situations.

## 3. Conclusions

The outline of a new, non-destructive method for the quantitative and fast measurement of FRET processes has been presented. Experiments will have to prove whether the method will be useful in praxi. The method is independent of any specific method of observation and may be applied to imaging as well as purely photometric methods. The method promises to be a useful tool, especially in high-resolution light microscopy.
